# Body size, swimming speed, or thermal sensitivity? Predator-imposed selection on amphibian larvae

**DOI:** 10.1186/s12862-015-0522-y

**Published:** 2015-11-02

**Authors:** Lumír Gvoždík, Radovan Smolinský

**Affiliations:** Institute of Vertebrate Biology AS CR, Květná 8, CZ 60365 Brno, Czech Republic

**Keywords:** Antipredator strategies, *Ichthyosaura*, Newts, Performance-fitness, Predator–prey interaction, Predator–prey size ratio, Selection differential, Selection experiment, Viability selection

## Abstract

**Background:**

Many animals rely on their escape performance during predator encounters. Because of its dependence on body size and temperature, escape velocity is fully characterized by three measures, absolute value, size-corrected value, and its response to temperature (thermal sensitivity). The primary target of the selection imposed by predators is poorly understood. We examined predator (dragonfly larva)-imposed selection on prey (newt larvae) body size and characteristics of escape velocity using replicated and controlled predation experiments under seminatural conditions. Specifically, because these species experience a wide range of temperatures throughout their larval phases, we predict that larvae achieving high swimming velocities across temperatures will have a selective advantage over more thermally sensitive individuals.

**Results:**

Nonzero selection differentials indicated that predators selected for prey body size and both absolute and size-corrected maximum swimming velocity. Comparison of selection differentials with control confirmed selection only on body size, i.e., dragonfly larvae preferably preyed on small newt larvae. Maximum swimming velocity and its thermal sensitivity showed low group repeatability, which contributed to non-detectable selection on both characteristics of escape performance.

**Conclusions:**

In the newt-dragonfly larvae interaction, body size plays a more important role than maximum values and thermal sensitivity of swimming velocity during predator escape. This corroborates the general importance of body size in predator–prey interactions. The absence of an appropriate control in predation experiments may lead to potentially misleading conclusions about the primary target of predator-imposed selection. Insights from predation experiments contribute to our understanding of the link between performance and fitness, and further improve mechanistic models of predator–prey interactions and food web dynamics.

**Electronic supplementary material:**

The online version of this article (doi:10.1186/s12862-015-0522-y) contains supplementary material, which is available to authorized users.

## Background

Most animals must avoid predation to survive and reproduce. Accordingly, prey has evolved diverse strategies to increase its chances of surviving a predator’s attack [1, 2]. Sufficiently mobile prey avoids predation primarily by fleeing, which should impose strong selection on escape performance. However, empirical evidence for predator-imposed selection on a key component of escape performance, maximum velocity, still remains relatively scarce (reviewed by [3, 4]), although the phenotype-performance-fitness paradigm [5] has dominated the field of ecological and evolutionary physiology for decades.

Finding the link between locomotor performance and fitness is complicated by, among other things, the highly integrative and plastic character of locomotion. In ectotherms, the maximum escape velocity is affected mainly by body size and temperature [6–8]. The role of body size in escape performance has been relatively well studied. Within a prey population, escape velocity scales positively with body size [9–11]. Despite its higher velocity, bigger prey is sometimes easier to catch by a predator than smaller individuals because of its size [12]. In addition, the ratio to predator size often determines the outcome of predator–prey interactions [13, 14].

In contrast to body size, a prey’s thermal sensitivity, i.e., the rate of change with the temperature of escape performance during predator encounters has received less attention. A recent theory offers a likely mechanism for predator-imposed selection on prey thermal sensitivity [15]. The thermal dependence of predator and prey performance traits is characterized by thermal performance curves that are typically left-skewed and unimodal [16]. Thermal performance curves may differ between both actors in their magnitude and rate, which determine their interaction dynamics [15, 17]. Accordingly, selection should favor a prey’s thermal rate (sensitivity) of escape performance that minimizes its predation under a range of environmental conditions. However, whether predators select for thermal sensitivity of maximum velocity remains virtually unknown.

Although field studies provide invaluable information about phenotypic selection in natural populations [18, 19], short-term selection experiments under laboratory or seminatural conditions allow better identification of a selective agent for a particular trait [20]. This is especially advantageous for performance traits, because prolonged viability selection experiments may be biased by plastic and life-history responses [21]. Among various predator–prey systems, short-term selection experiments on prey swimming capacity have been frequently realized using dragonfly and amphibian larvae. Although some studies corroborated the importance of maximum swimming velocity during predation episodes [22–25], other findings are equivocal. In some species, tadpole’s survival depends on its ability to attract a predator’s attention to the tail, a relatively expendable body part, rather than maximum swimming velocity [26–28]. In addition, while tadpoles are often exposed to thermally varying conditions in the field, most studies have been performed under constant temperature or unknown conditions, which may hide the primary target of predator-mediated selection.

Here, we examined the influence of predation on a prey’s body size, absolute and relative maximum velocity, and its thermal sensitivity, using replicated short-term selection experiments under seminatural conditions. Unlike previous studies on tadpoles, we chose newt larvae as prey, because they are more sedentary than tadpoles of Central European taxa and respond to predator (dragonfly larva) encounters by burst swimming. Swimming speed is size-dependent in amphibian larvae, and thus we predicted that bigger and faster larvae will survive a predator’s attack more often than smaller and slower individuals [25]. In addition, because newt larval swimming velocity is influenced by acute thermal conditions more than a dragonfly larvae’s prey capturing mechanism [29, 30], we predicted that larvae achieving high swimming velocities across ecologically relevant temperatures will have a selective advantage over more thermally sensitive individuals.

## Methods

### Study species

*Ichthyosaura alpestris* (Laurenti, 1768) is a medium-sized newt (total length [TL] of up to 12 cm) commonly distributed across Western and Central Europe. It usually has a biphasic lifestyle with an aquatic and terrestrial period. In Central Europe, the aquatic breeding period typically lasts from April until June. Larvae hatch during May and usually metamorphose during summer. *Aeshna cyanea* (O. F. Müller, 1764) is a common dragonfly species distributed throughout most of Europe. In our study population, the larval period lasted two years. Dragonfly larvae are visually-oriented predators. They prey on various aquatic invertebrates and amphibian larvae. *Aeshna cyanea* larvae are a frequent predator of *I. alpestris* larvae [31].

All experimental procedures were approved by the Departmental Committee of the Academy of Sciences of the Czech Republic (research protocol no. 14/2013) and comply with the current laws of the Czech Republic. The Environmental Department of the Regional Authority of Vysočina, Czech Republic, issued the permission to capture newts (KUJI 224/2013).

### General maintenance

To fulfill the aims of this study, we reared newt larvae and dragonfly larvae under seminatural conditions. In sampled newt larvae, we measured their body size and swimming velocity at two temperatures. Following predation trials, we re-measured body size and swimming velocity in survived larvae.

Eggs (*n* ≈ 2000) from ten female newts were collected from aquatic plants growing in ten separate outdoor mesocosms following published protocols [32]. Ten rearing tanks (90 × 63 × 47 cm high) initially filled with 100 L of well water were randomly arranged outdoors under full-sun conditions. Previous measurements showed that newts in tanks experienced water temperatures similar to those in natural pools [32]. The tank water temperatures (bottom [mean ± SD]: 14.6 ± 4.3 °C; surface: 16.1 ± 4.0 °C) and surface light intensity (13.9 ± 25.9 klx) were recorded at hourly intervals using dataloggers (resolution 0.5 °C; DS1921G-F5, Maxim Integrated Products, Sunnyvale, CA, USA; HOBO UA-002-08, Onset, Bourne, MA, USA). After female removal, the number of eggs was manipulated to attain a similar starting larval density (approx. 0.5 larvae L^−1^) in each tank. Although this number was higher than those reported in literature for older larvae (0–100 individuals m^2^ [31]), given the typical larval survivorship pattern [33], we assumed that the starting density was an ecologically realistic estimate for our study population. Living plankton was added to tanks every second day to provide *ad libitum* food for the developing larvae.

Overwintered dragonfly larvae (*n* = 60) were captured from the same pools as adult newts (see above). Dragonfly larvae groups (*n* = 5 per group) were placed in stock tanks, which were plastic aquaria (50 × 30 × 18 cm high) filled with 18 L of well water. Each aquarium was equipped with ample aquatic vegetation (*Egeria densa*) to provide shelter for larvae. Aquaria were randomly placed outdoors among tanks with newt larvae, except during predation experiments (see below). Dragonfly larvae were fed with plankton, *Chironomus* larvae, and *Tubifex* worms at three day intervals.

In addition to predation trials (see below), we used ten dragonfly larvae in rearing tanks to induce a plastic response in newt larvae [31, 34]. One caged dragonfly larva (≈5 cm total length) was added to each tank. Because dragonfly larvae were frequently found in our study populations, the presence of predator cues was necessary to obtain ecologically realistic phenotypes of newt larvae for predation experiments. Dragonfly larvae were individually placed in perforated floating tubes (1 L) that contained a piece of *E. densa* as a perch. To provide predation cues for the developing newt larvae, dragonfly larvae were fed with living newt larvae, at three-day intervals. Dragonfly larvae were rotated randomly among tanks in weekly intervals.

### Swimming performance trials

Swimming velocity was measured in newt larvae before and after predation and in control trials (see below). Haphazardly captured larvae were individually placed in Petri dishes (8 cm diameter) at experimental temperatures (10 or 20 °C) at least two hours before trials. The order of temperatures was randomly selected for each individual. Experimental temperatures were chosen according to water temperatures that newt larvae frequently experience in their native habitat [35]. Swimming trials were realized in a walk-in climatic chamber, which guaranteed stable temperature conditions during measurements.

Swimming performance trials were performed by one person (RS) following the experimental protocol by [30]. Newt larvae were placed in the middle of a circular arena (30 cm diameter) filled with water up to 1 cm. Its escape response was induced by gentle touching of its tail with a fine stainless steel probe. Each larva was stimulated four times at one temperature before and after a predation episode. Swimming bouts were recorded using a digital video camera (frame frequency 50 Hz; Panasonic NV-GS500, Matsushita Electric Industrial, Osaka, Japan) mounted perpendicularly above the arena. To obtain sharp contours of swimming larva, lighting (four 8 W fluorescent tubes) was provided through the semitransparent bottom. Larvae rested at least three hours between successive trials at different temperatures.

Video records were processed using motion analysis software (MaxTraq, Innovision Systems, Columbiaville, MI, USA). The maximal distance traveled during 0.02 s, averaged from two successive frames, was used for the calculation of the maximum swimming velocity for each larva. Each swimming trial was subjectively judged as good or bad. Bad trials (4 %), e.g. swimming along the walls of arena, were discarded from further analyses.

Total length (tip of snout to end of tail) was measured from the digital images of still larvae using TpsDig software (version 2.02; [36]. To attain the highest accuracy, TL was averaged from three consecutive measurements. All measurements (resolution 0.001 cm) were performed by one person (RS).

We used *Q*_10_ rate to characterize thermal sensitivity of swimming velocity between 10 and 20 °C before and after predator-imposed selection: *Q*_10_ = *U*_20_/*U*_10_, where *U*_20_ and *U*_10_ are maximum swimming velocities at 20 ° and 10 °C, respectively. Accordingly, the thermal dependence of swimming velocity was characterized by the rate, *Q*_10_, and the magnitude, *U*_max_, which is the mean maximum velocity across temperatures (*U*_max_ = (*U*_10_ + *U*_20_) / 2).

### Predation trials

We performed short-term predation trials following the experimental protocol by Smolinský and Gvoždík [32]. We used plastic aquaria (18 L) filled with tap water and inoculated with plankton-containing pond water (0.5 L). To avoid the confounding influence of habitat complexity [37], each aquarium contained only a string (30 cm) of *E. densa*, which provided a perch for the dragonfly larva, and a datalogger (see above) that recorded water temperatures and light intensity at hourly intervals. Aquaria were covered with a fine mesh. In total, we used 40 aquaria distributed outdoors across various light conditions.

We placed randomly chosen newt larvae (*n* = 10) at the same developmental stage (the fifth toe clearly visible) into an aquarium for 12 h prior to the beginning of an experiment (08:00 h). The used larval density (67 individuals m^−2^) was within the range of ecologically realistic values [31]. We then added one randomly chosen dragonfly larva (total length [mean ± SD] = 3.8 ± 0.5 cm) from rearing tanks into the aquaria, and left it undisturbed for 24 h. The relatively short duration of the selection episode was chosen to eliminate confounding factors on prey escape velocity, such as developmental and plastic responses, and to prevent eradication of the whole group by a predator. Dragonfly larvae were starved for three days between successive trials to control for the different hunger levels among them. The larvae were used repeatedly (2–4 times) for predation trials. Each predation trial was spatially paired with a control without a predator. The presence of a control allowed us (i) to eliminate the confounding effect of spatiotemporal environmental variation and larval training on selection differentials, and (ii) to estimate the repeatability of measured traits at a group level before and after trials. The number of predation trials per day varied depending on the availability of final-stage larvae in a given tank. The whole experimental period lasted six weeks (mid June–end of July).

To standardize results from predation and control trials, i.e. means with various SDs, we calculated standardized linear and non-linear selection differentials [18, 38, 39] for TL, *U*_max_, size-adjusted *U*_max_ (residuals from the linear relationship of *U*_max_ on TL; *U*_rel_; see Additional file 1), and *Q*_10_ as follows:

1 $$ {S}_i=\frac{{\overline{z}}_i after-{\overline{z}}_i before}{s_i before} $$

where *S*_*i*_ is the standardized linear selection differential of trait *i*, ${\overline{z}}_i$ is the mean of *i* before and after the viability selection episode, and *s*_*i*_, is the standard deviation of *i*;

2 $$ {C}_i=\frac{s_i^2 after-{s}_i^2 before}{s_i^2 before}+{S}_i^2 $$

where *C*_*i*_ is the standardized nonlinear selection differential after adjusting for directional selection and *s*_*i*_^2^ is the variance of *i* before and after the viability selection episode;

3 $$ {C}_{i,j}=\frac{s_{i,j} after-{s}_{i,j} before}{s_{i,j} before}+{S}_i{S}_j $$

where *C*_*i,j*_ is standardized nonlinear selection differential for correlation selection on two traits, *i* and *j*, after adjusting for the effect of directional selection, *s*_*i,j*_ is covariance between *i* and *j*. All traits but *U*_rel_ were transformed to a mean zero before calculations of selection differentials [38].

### Statistical analyses

We visually checked data (means, variances, and covariances per predation trial aquarium) to meet the assumptions of parametric tests. Because of a relatively low sample size and the presence of outliers, which lacked an objective reason for their deletion, we applied a randomization approach for further analyses. We applied the Spearman permutation test to examine correlations between traits in control trials. Selection differentials between predation treatment and controls were compared using a permutation test (9999 permutations) for paired data. Confidence intervals (95 %) for means were estimated using a non-parametric bootstrapping procedure (9999 replications). The effect of predator size and temperature variation during predation trials on standardized selection differentials was examined using permutation multiple regression. Because temperature fluctuation is associated with both mean temperature and light intensity [32], we chose this variable as a representative measure of environmental variation. Statistical analyses were performed using the ‘coin’ [40] and ‘boot’ [41] libraries in R [42] and ‘PERMANOVA’ package for PRIMER (version 6; Primer E, Lutton, UK).

## Results

We performed 30 pairs of predation and control trials using 600 newt larvae. Water temperatures experienced by larvae during a predation episode varied among trials (mean = 18.9 ± 4.0 °C; range = 8.8 ± 3.8 °C). After 24 h, larval mortality ranged between 0 and 0.5 (mode = 0.2). Three trials with zero mortality (i.e. no selection) and their corresponding controls were discarded from further analyses. All newt larvae from the control treatment experienced zero mortality.

**Fig. 1:**
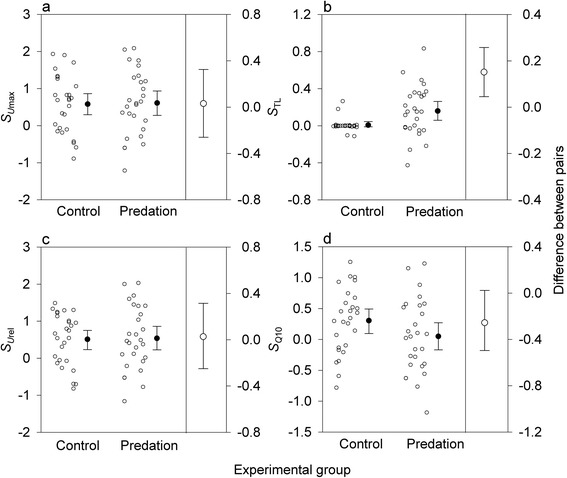
Linear selection differentials for prey traits. Linear selection differentials (*S*_i_) for (**a**) maximum swimming velocity (*U*_max_), (**b**) total length (TL), (**c**) size-corrected *U*_max_ (*U*_rel_), and (**d**) thermal sensitivity of *U*_max_ (*Q*_10_) in newt larvae subjected to predator-imposed selection episodes and controls. The right *y*-axis shows the magnitude (mean ± 95 % CIs) of paired differences between predation and control groups. Datapoints are jittered horizontally to reduce overlap. Group means are with 95 % CIs

Non-zero linear selection differentials were found in *U*_max_, *U*_rel_, and TL (Fig. 1). However, only *S*_TL_ was higher relative to its control (*Z* = 2.92, *P* = 0.002; *S*_*U*max_: *Z* = 0.246, *P* = 0.81; *S*_*U*rel_: *Z* = 0.218, *P* = 0.83). In *Q*_10_, selection differentials in the control group were higher than in the predation group (*Z* = 2.271, *P* = 0.02).

**Fig. 2:**
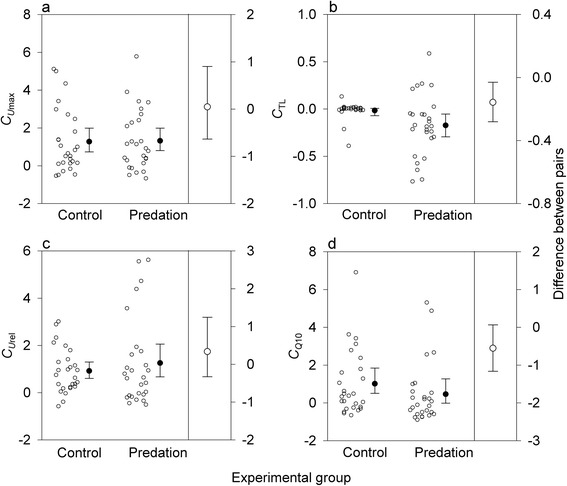
Nonlinear univariate selection differentials. Nonlinear univariate selection differentials (*C*_i_) for (**a**) maximum swimming velocity (*U*_max_), (**b**) total length (TL), (**c**) size-corrected *U*_max_ (*U*_rel_), and (**d**) thermal sensitivity of *U*_max_ (*Q*_10_) in newt larvae subjected to predator-imposed selection episodes and controls. See Fig. 1 for further details

**Fig. 3:**
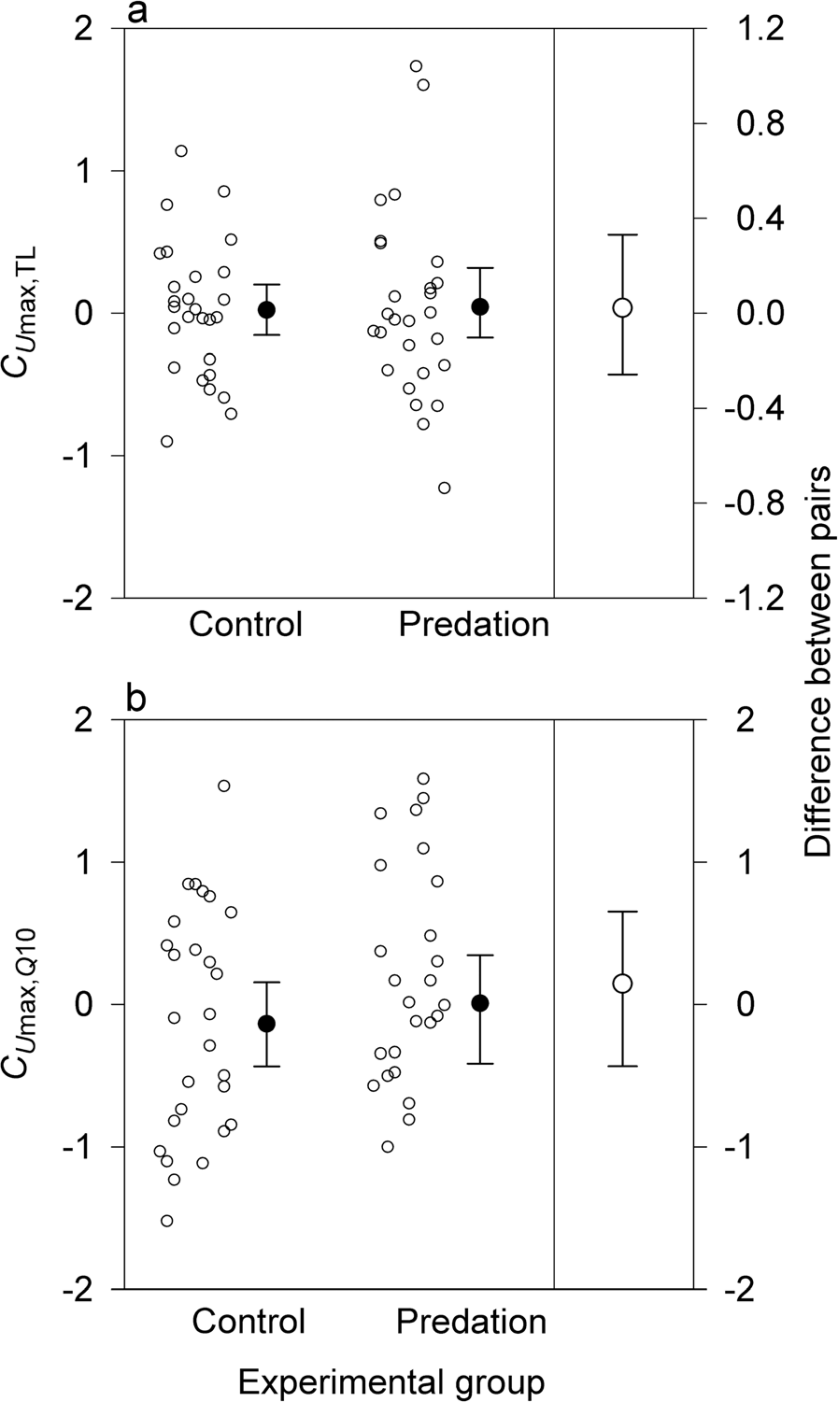
Nonlinear bivariate selection differentials. Nonlinear bivariate selection differentials (*C*_i,j_) for (**a**) maximum swimming velocity (*U*_max_) and total length (TL), and (**b**) *U*_max_ and thermal sensitivity of *U*_max_ (*Q*_10_) in newt larvae subjected to predator-imposed selection episodes and controls. See Fig. 1 for further details

Except for TL (*Z* = 2.67, *P =* 0.006), nonlinear univariate selection differentials were similar between predation and control groups in all traits (*C*_*U*max_: *Z* = 0.14, *P* = 0.89; C_*U*rel_: *Z* = 0.97, *P* = 0.33; *C*_*Q*10_: *Z* = 1.93, *P* = 0.05; Fig. 2). Bivariate nonlinear selection differentials provided no support for correlated selection on *U*_max_ and *TL* (*Z* = 0.17, *P* = 0.86; Fig. 3) or *U*_max_ and *Q*_10_ (*Z* = 0.64, *P* = 0.53). Predator size and temperature explained very little variation in the selection differentials of all traits (Table 1).

**Table 1:** Influence of predator size and water temperature variation on selection differentials of prey traits

Selection differential	Effect of predator size		Effect of temperature variation	
	Pseudo-*F*_2,25_	*P*	Pseudo-*F*_2,25_	*P*
*S*_*U*max_	1.11	0.30	0.75	0.33
*S*_TL_	0.35	0.56	3.49	0.08
*S*_*U*rel_	0.94	0.35	0.43	0.51
*S*_*Q*10_	0.08	0.77	0.20	0.66
*C*_*U*max_	0.53	0.48	0.02	0.88
C_TL_	0.92	0.35	0.16	0.70
*C*_*U*rel_	<0.01	0.96	0.15	0.70
*C*_*Q*10_	<0.01	0.98	0.20	0.90
C_*U*max,TL_	0.35	0.56	1.47	0.24
C_*U*max,*Q*10_	2.96	0.09	0.04	0.85

Results were obtained using a permutation multiple regression model. *U*_max_, maximum swimming velocity, *TL* total length, *U*_*rel*_ size-corrected *U*_max_, *Q*_10_, thermal sensitivity of *U*_max,_ *S*_*i*_ linear selection differential, *C*_*i*_ univariate nonlinear selection differential, *C*_i,j_ bivariate nonlinear selection differential

**Fig. 4:**
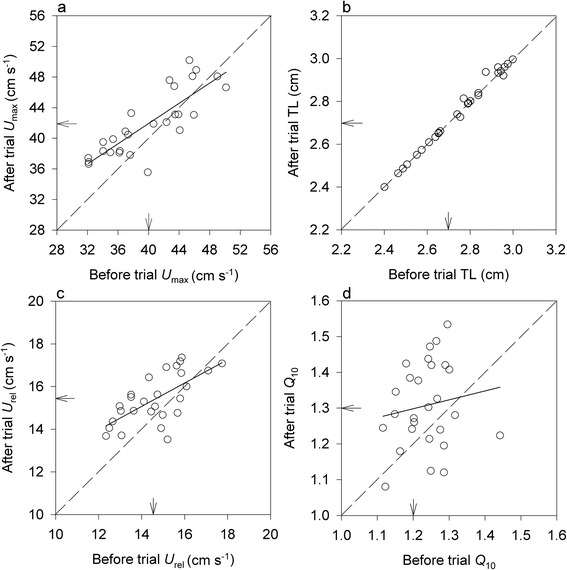
Associations between repeated measurements before and after a control trial. **a** Maximum swimming velocity (*U*_max_), (**b**) total length (TL), (**c**) size-corrected *U*_max_ (*U*_rel_), and (**d**) thermal sensitivity of *U*_max_ (*Q*_10_). Datapoints are group means. Except (**b**), datapoints are fitted using linear regression to show trends. Dashed lines indicate 100 % repeatability. Arrows denote overall means

In control aquaria, mean *U*_max_, TL, and *U*_rel_ were positively associated before and after trials (*U*_max_: *r* = 0.84, *P* < 0.001; TL: *r* = 0.99, *P* < 0.001; *U*_rel_: *r* = 0.77, *P* < 0.001; Fig. 4). Correlation of *Q*_10_ means was statistically non-significant (*r* = 0.14, *P* = 0.51) suggesting low repeatability of this trait. Except for well-reproducible TL means (*Z* = 0.23, *P* = 0.75), larvae swam faster and were more thermally sensitive after than before the control trial (*U*_max_: *Z* = 3.79, *P* < 0.001; *U*_rel_: *Z* = 3.77, *P* < 0.001; *Q*_10_: *Z* = 3.17, *P* < 0.001; Fig. 4).

## Discussion

A key assumption of locomotor performance studies is selective advantage of maximum velocity. In addition, the recent theory of thermally sensitive predator–prey interactions [15] implies selection not only on the magnitude of prey escape velocity but also on its rate of response to temperature. Our results provide no support for these notions. While non-zero selection differentials might suggest predator-imposed selection on prey *U*_max_, *U*_rel_, and TL, their comparison with the control revealed selection only on TL. Repeated measurements of *U*_max_ and *Q*_10_ in control trials revealed their low group repeatability, which contributed to the non-detectable selection on both traits.

The thermal sensitivity of prey swimming velocity was unaffected by predation. In fact, *S*_*Q*10_ estimates suggest that selection for *Q*_10_ was even higher in the control (zero mortality) than in the predation group. The likely explanation for this apparent artifact is the poor group repeatability of *Q*_10_, which resulted from low *Q*_10_ values between 10–20 °C (1.3 ± 0.1) and a relatively high variation in velocity measures. Interestingly, the low *Q*_10_ values across the 10–20 °C range are partially caused by a plastic response to fluctuating thermal conditions [30]. Although *Q*_10_ appears negligible for larval escape success, it cannot be ruled out that the plastic response towards low thermal sensitivity was shaped by selection that occurred in the past. Low repeatability and selection on thermal sensitivity rather than on absolute performance values [43] should be considered among potential factors slowing the evolution of thermal performance curves for maximum velocity.

Positive linear selection differentials of TL suggest that dragonfly larvae preferably preyed on small newt larvae. Accordingly, predation reduced the variance of TL, which resulted in negative nonlinear selection differentials of this trait. Size-selective predation has been widely documented in amphibian larvae [44–46]. Why dragonfly larvae largely preyed on small newt larvae is unknown. Theory predicts that predator attack rate depends on the predator–prey size ratio ([47], but see [48]). Although the size (TL) ratio approached unity (1.3 ± 0.2), the maximum TL of newt larvae provides no mechanic limitation for dragonfly larvae to capture and subdue their prey ([49]; R. Smolinský, personal observations; but see [50]). In addition, predator size had a negligible influence on *S*_TL_ suggesting that results were little affected by the predator–prey size ratio or predator size correlates, such as encounter rate and handling time.

Alternatively, predator-imposed selection on prey body size was mediated by its correlation with other traits. Contrary to this notion, bivariate selection differentials indicate that predator-imposed selection acted independently on TL and *U*_max_ in newt larvae. The same result has been reported in tadpoles [25] suggesting independent selection on these traits in both systems. Given the common occurrence of correlational selection [51], this result is a little bit surprising. Among other possible correlates of body size, motor activity (foraging activity or time spent swimming) appears the major determinant of predator-mediated mortality in amphibian larvae [52–54], including the species studied [55]. However, theory predicts that more active individuals grow faster because of higher food uptake, and thus they are bigger than more sedentary counterparts [56]. If the mortality-growth trade-off [57] holds in newt larvae, odonate predators would preferably prey on bigger individuals in experimental arenas, which contradicts our findings. In addition, hungry dragonfly larvae may prey on tadpoles irrespective of their activity level ([58], but see [26, 59]). Hence, identifying determinants of larval escape success requires further research.

Newt larvae swam faster after than before a predation trial, which produced positive *S*_*U*max_ values. However, their comparison with controls revealed zero difference, which implies that the shift in *U*_max_ is caused by other factors than predation. The motivation to perform at maximum speed varies between field and laboratory conditions [60] or between natural and artificial stimuli [61, 62]. In addition, previous experience and training may contribute to the shift in swimming velocity. In contrast to the shifted means, the paired velocity measures were highly-correlated at a group level suggesting good repeatability of this trait (see also [61]). It follows that the ability to obtain the same group mean is more important than the strength of association between repeated values [63, 64] in predator-imposed selection studies.

## Conclusions

Although swimming velocity remains a popular measure of whole-animal performance, this trait played a less important role in newt larvae than has been previously thought. While earlier studies demonstrated the relationship between tail morphology and swimming performance [65], and between tail morphology and survival [31], our study failed to provide the missing link between swimming velocity and survival. This suggests that predator-induced plasticity in the tail area serves another purpose than to improve the escape performance, perhaps to lure predator’s attack to the dispensable body part as in tadpoles [66]. Given the mixed empirical support across taxa (see Background), the locomotor performance-fitness assumption always requires experimental verification in a focal species. From an ecological view, the key role of body size in escaping predation appears important in both tadpoles (see Background) and newt larvae (this study; see also [46]) despite their disparate foraging strategies, grazing and ambush predation, and accordingly motor activity levels. A potentially important but largely overlooked consequence of size-selective predation is its contribution to the thinning effect of predation [67, 68], i.e. the body size increase of survived prey may result not only from reduced competition but also from preferential predation on small prey. Finally, our study showed the necessity of well-replicated and controlled predation experiments to properly parameterize mechanistic models of predator–prey interactions and food web dynamics.

### Availability of supporting data

The data set supporting the results of this article is available in the Dryad Digital Repository, http://dx.doi.org/10.5061/dryad.vh783 [69].

## Additional file

Additional file 1:
**Maximum swimming velocity as a function of total length in newt larvae.** Velocity values are means across temperatures (10 °C and 20 °C). Dashed lines denote 95 % confidence intervals. *y* = 15.28 + 8.98*x*; *R* ^2^ = 0.24. (PDF 66 kb)

## Abbreviations

*C*_i_: Univariate nonlinear selection differential
*C*_i,j_: Bivariate nonlinear selection differential
*Q*_10_: Thermal sensitivity of *U*_max_
*S*_i_: Linear selection differential
TL: Total length (snout tip to tail tip)
*U*_10_: Maximum swimming velocity at 10 °C
*U*_20_: Maximum swimming velocity at 20 °C
*U*_max_: Mean maximum swimming velocity across 10–20 °C range
*U*_rel_: Size-corrected *U*_max_
