# Supplementary figures and images for: Body size, swimming speed, or thermal sensitivity? Predator-imposed selection on amphibian larvae
Source: BMC Evol Biol. 2015 Nov 2;15:238. doi: 10.1186/s12862-015-0522-y (PMC4630873; doi:10.1186/s12862-015-0522-y)

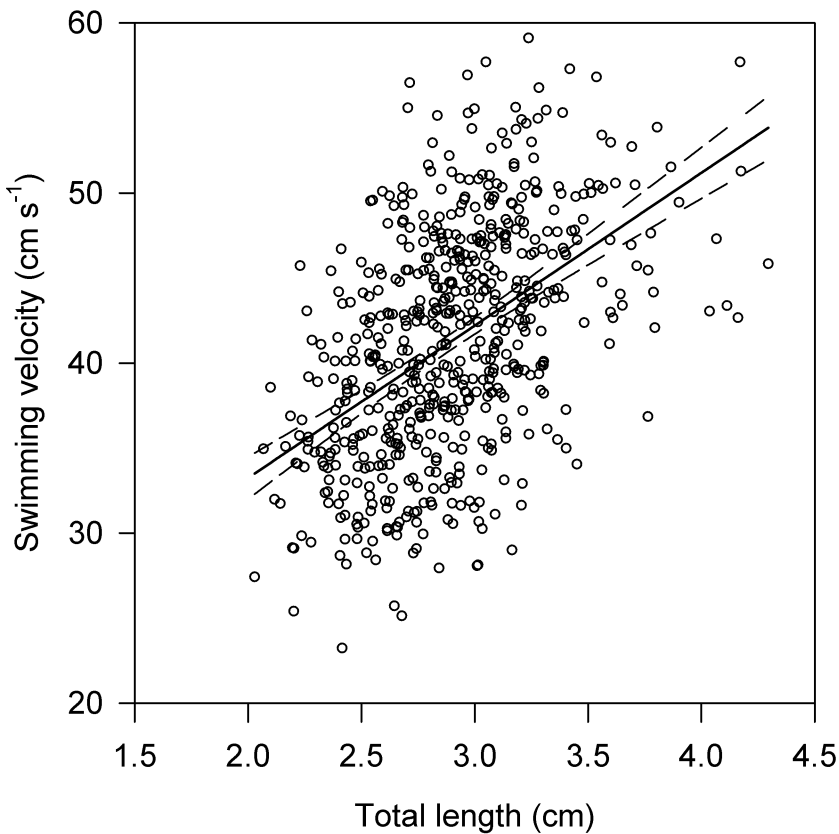

Supplement: Additional file 1: — Maximum swimming velocity as a function of total length in newt larvae. Velocity values are means across temperatures (10 °C and 20 °C). Dashed lines denote 95 % confidence intervals. y = 15.28 + 8.98x; R 2 = 0.24. (PDF 66 kb) [file 12862_2015_522_MOESM1_ESM.pdf]
